# Cohort Description of the Madagascar Health and Environmental Research–Antongil (MAHERY–Antongil) Study in Madagascar

**DOI:** 10.3389/fnut.2019.00109

**Published:** 2019-07-19

**Authors:** Christopher D. Golden, Cortni Borgerson, Benjamin L. Rice, Lindsay H. Allen, Evelin Jean Gasta Anjaranirina, Christopher B. Barrett, Godfred Boateng, Jessica A. Gephart, Daniela Hampel, Daniel L. Hartl, Erwin Knippenberg, Samuel S. Myers, Dera H. Ralalason, Herlyne Ramihantaniarivo, Hervet Randriamady, Setareh Shahab-Ferdows, Bapu Vaitla, Sarah K. Volkman, Miadana Arisoa Vonona

**Affiliations:** ^1^Department of Nutrition, Harvard T.H. Chan School of Public Health, Boston, MA, United States; ^2^Department of Environmental Health, Harvard T.H. Chan School of Public Health, Boston, MA, United States; ^3^Madagascar Health and Environmental Research, Maroantsetra, Madagascar; ^4^Department of Anthropology, Montclair State University, Montclair, NJ, United States; ^5^Department of Organismic and Evolutionary Biology, Harvard University, Cambridge, MA, United States; ^6^ARS Western Human Nutrition Research Center, United States Department of Agriculture, Davis, CA, United States; ^7^Department of Nutrition, University of California, Davis, Davis, CA, United States; ^8^CH Dyson School of Applied Economics & Management, Cornell University, Cornell, NY, United States; ^9^National Center for Socio-Environmental Synthesis (SESYNC), Annapolis, MD, United States; ^10^Cooper/Smith, Washington, DC, United States; ^11^Service de District de la Santé Publique de Maroantsetra, Ministère de la Santé Publique d'Analanjirofo, Maroantsetra, Madagascar; ^12^Direction Generale d'Antananarivo, Ministère de la Santé Publique, Antananarivo, Madagascar; ^13^Department of Immunology and Infectious Diseases, Harvard T.H. Chan School of Public Health, Boston, MA, United States; ^14^Infectious Disease Initiative, Broad Institute of MIT and Harvard, Cambridge, MA, United States; ^15^College of Natural, Behavioral, and Health Sciences, Simmons University, Boston, MA, United States

**Keywords:** food security, micronutrient deficiencies, fisheries, malnutrition, health impact assessment, seafood

## Abstract

The Madagascar Health and Environmental Research-Antongil (MAHERY-Antongil) study cohort was set up in September 2015 to assess the nutritional value of seafood for the coastal Malagasy population living along Antongil Bay in northeastern Madagascar. Over 28 months of surveillance, we aimed to understand the relationships among different marine resource governance models, local people's fish catch, the consumption of seafood, and nutritional status. In the Antongil Bay, fisheries governance takes three general forms: traditional management, marine national parks, and co-management. Traditional management involves little to no involvement by the national government or non-governmental organizations, and focuses on culturally accepted Malagasy community practices. Co-management and marine national parks involve management support from either an non-govermental organization (NGO) or the national government. Five communities of varying governance strategies were enrolled into the study including 225 households and 1031 individuals whose diets, resource acquisition strategies, fisheries and agricultural practices, and other social, demographic and economic indicators were measured over the span of 3 years. Clinical visits with each individual were conducted at two points during the study to measure disease and nutritional status. By analyzing differences in fish catch arising from variation in governance (in addition to intra-annual seasonal changes and minor inter-annual changes), the project will allow us to calculate the public health value of sustainable fisheries management approaches for local populations. There is hope that coastal zones that are managed sustainably can increase the productivity of fisheries, increasing the catch of seafood products for poor, undernourished populations.

## Introduction

The Madagascar Health and Environmental Research-Antongil (MAHERY-Antongil) study cohort was set up in September 2015 to assess the nutritional value of seafood for the coastal Malagasy population living along Antongil Bay in northeastern Madagascar. We aimed to understand the relationships among different marine resource governance models, local people's fish catch, the consumption of seafood, and nutritional status (1–3).

Marine conservation has been hypothesized to increase fisheries productivity and to increase the catch of seafood for local consumption (4–6). The direct impacts of fisheries management on human nutrition have been relatively understudied, however, with many long-lasting policy narratives lacking a rigorous evidence base (1, 7). Several studies have created a foundation to assess this relationship [e.g., (3, 8)]. Certain studies have examined the contribution of fish to overall dietary patterns [e.g., (9, 10)], and in some cases to nutrient intake, including micronutrients, vitamins, and protein [e.g., (11)] or linking to nutritional status as proxied by anthropometry [e.g., (12, 13)]. Our study differs in that it connects fisheries management practices to dietary patterns, nutrient intakes, anthropometric measures, and clinical biomarkers of micronutrient, vitamin, and fatty acid status.

Globally, beginning in the 1980s, decentralized management of marine systems and coastal zones became a preferred way to co-manage environments joining local communities with engagement and support from an non-govermental organization (NGO) (14, 15). This structure of governance is often called a locally managed marine area (LMMA). LMMAs devolve centralized government control over coastal zones to local authorities. In theory, they allow local communities to prevent overexploitation and reinforce sustainable management by tapping their unsurpassed knowledge of the local resource and ability to monitor human activity to assert control over their own resources. The goal of LMMAs is to simultaneously protect biodiversity and increase fisheries productivity in support of local human health and livelihoods. Communities perceive that LMMAs confer benefits (16), and we sought to assess whether these governance systems provide benefits to human nutrition and health. Marine conservation and fisheries management improvements could be viewed as a public health nutrition intervention; targeted conservation efforts can rehabiliate fish stocks and increase fisheries productivity, including reef systems in Madagascar (17), potentially creating synergies between marine conservation, and human well-being.

Using this variation in fisheries management, we formed the cohort to understand the contribution of wild captured seafood in supporting the nutritional well-being of local people. The cohort also provided an opportunity to understand the interactive dynamics among subsistence fishing, dietary intake, nutritional status, and the incidence of intestinal parasites and malaria. To account for seasonal changes in dietary patterns and disease status, our team characterized the diets of individuals over long time scales, similar to past MAHERY cohort studies (18). In the context of the very high prevalence of stunting (linear growth retardation) in Madagascar—over 50% of children under 5 years of age are stunted (19)—understanding the relationship of diets to physical, nutritional and developmental outcomes across the lifecourse is critical. This is particularly the case in low-income countries, where deficiencies of iron, zinc, and vitamins A, D, and B vitamins (especially B12) cause a range of poor health outcomes, including increased morbidity and mortality, poor child development and pregnancy outcomes, and low micronutrients in maternal milk (20, 21).

## Methods and Analysis

### Study Site

The Antongil Bay of northeastern Madagascar is characterized by lowland and litoral rainforest, a high prevalence of malnutrition, and a human population of rural agriculturalists and fisherfolk predominantly of Betsimisaraka ethnicity. We selected this study site for two primary reasons: (1) it is a coastal population with heavy subsistence reliance on local seafood; and (2) it offers the opportunity to study varying marine resource management systems. Market access is limited and domesticated meats and aquaculture products are an infrequent luxury in this region (22, 23). Thus, the consumption of seafood from wild capture fisheries using nets, lines, and shore gleaning, can provide crucial micronutrients that are otherwise unavailable in the diet (2).

In the Antongil Bay, fisheries governance takes three general forms: traditional management, marine national parks, and co-management. Traditional management involves little to no involvement by the national government or non-governmental organizations, and focuses on culturally accepted Malagasy community practices that tend not to restrict fishing gear or protect certain locations from harvest. In terms of formal restrictions on capture, traditional management tends to be the least strict of the three management forms. Marine national parks are managed by the national government and often involve “no-take zones” enforced by local government agents; they are the strictest form of fisheries management. Co-management is often enabled through financing and training by a NGO partner. The Wildlife Conservation Society (WCS) has been implementing co-management efforts through a series of LMMAs. We hypothesized that co-management would lead to greater subsistence catch and more consumption of seafood in comparison to traditionally managed communities and communities adjacent to a marine national park. This hypothesis is rooted in evidence from the Pacific that found that periodically harvested closures can maximize harvest efficiency and fisheries yield beyond that achievable by no-take permanent closures or no management (24).

### Study Population

In 2015, we began a prospective cohort study in five remote rural communities (hereafter referred to as Communities 1–5 or C1–5) along the coast of Antongil Bay in northeastern Madagascar. The fisheries of Communities 1 and 2 are traditionally managed without governmental or NGO oversight, co-management, or control. The fisheries of Communities 3 and 4 are LMMAs co-managed by WCS since 2007. Community 5 is located near a marine national park (officially designated in 1997), where the national government installed a protected area strictly limiting access to the local fishery. In terms of exclusive access to fishing rights, Community 5 had exclusive “sea tenure”— no other communities can fish inside the marine national park—whereas Communities 1–4 did not have sea tenure, and fishing from adjacent villages was common.

**Figure 1:**
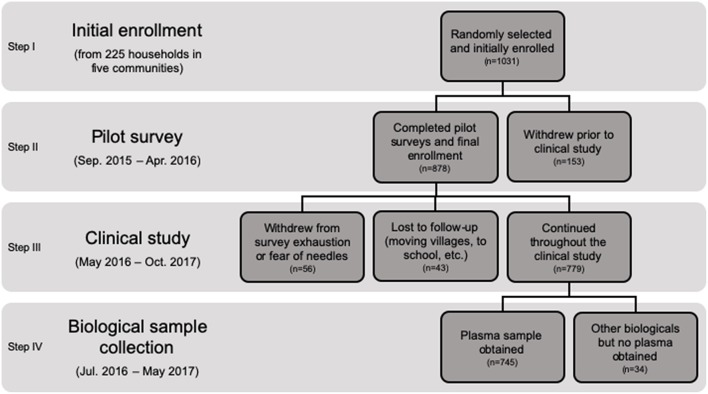
Consort figure of the MAHERY-Antongil study population.

The cohort in Communities 1–5 was maintained from September 2015 until October 2017. Using a community-wide comprehensive census that we created between May to June 2015, we assigned each household a number and randomly selected households to be included in the research. The C1 sample contained 25 households (out of a total of 360 households), and C2–5 contained 50 households each (out of a total of 260, 634, 180, and 98 households, respectively). The C1 household sample was less because C1 only had a half-time local assistant to monitor the population, whereas C2-5 had full time assistants present to assist with the research. At several points, households in the initial enrollment were lost. First, we lost several households in the period between initial enrollment and the start of our pilot period, largely due to some households moving outside the study area. These households were replaced through randomization prior to beginning the pilot period. The initial pilot period (from September 2015 until April 2016) began with 225 households and a total of 1,031 individuals, during which we finalized enrollment and began collecting preliminary baseline survey information. During this pilot period, 35 of the initial 225 households withdrew and we went from 1,031 to 878 individuals (Figure 1). Repeated socio-economic and dietary surveys of the cohort of 878 individuals were conducted prior to beginning the survey portion of the clinical study in May 2016 and well before our first collection of clinical samples between July–August 2017. Following final enrollment, we had ~6.4% of the overall clinical enrollment subjects withdraw due to survey fatigue and/or fear of needles and 4.9% of the enrollment was lost to follow-up due to moving villages or absence due to attending school in another area. Our final sample population included 779 individuals of both sexes from birth to 91 years of age that enrolled and completed the clinical aspects of the study.

### Data Collection

**Figure 2:**
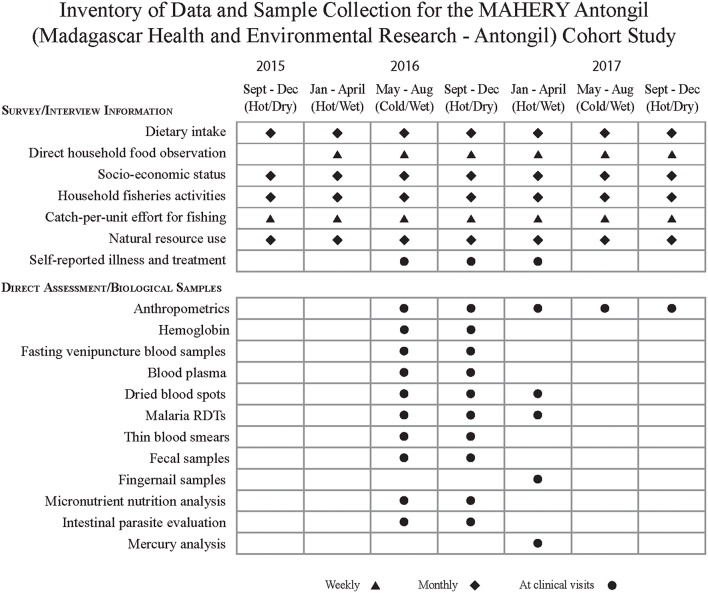
Data and samples collected during the MAHERY-Antongil cohort study.

Throughout the 28 month study, our team used a mixed-methods approach to collect a variety of information spanning social, environmental, and clinical health data (Figure 2). We surveyed all individuals in the study once per month over the duration of the study. For children who were too young to answer their own questions (typically under 5 years of age), we accepted proxy responses from primary caregivers.

#### Socio-Economic Status

We assessed socio-economic status through: (1) weekly recalls of each household's food-related *expenditures* (this did not include the consumption of home-grown foods); and (2) monthly recalls of (a) the amount (and source) of all cash *income* earned by members of each household, (b) the number of select commercial *assets owned* by members of each household (e.g., motorcycles, bicycles, radios, laptops/tablets, flashlights, fishing nets, boats, livestock), and (c) the amount of “luxury” *goods consumed* or used by each household during the prior day, week, and/or month (e.g., sugar, coffee, oil, salt, petrol). At the start of the study, we also recorded the primary economic activities of each household, their access to primary and secondary education, medical care, potable water, public safety services (e.g., police officers, court), and a list of market goods available from local stores. On a monthly basis, we conducted a morbidity recall to estimate the incidence of certain types of illnesses.

#### Dietary Intake

We measured dietary intake through monthly 24-h and prior-week recall assessments and food frequency surveys. These permit the calculation of the frequency, seasonality, and diversity of diets, though not the volume of foods or nutrients consumed. To estimate weights, we visited the same six households in each community, without prior notice, once per week and observed them during breakfast, lunch, and dinner to determine approximate weights of foods that were recorded in the dietary intake qualitative assessments. Prior to cooking, a research assistant from the community measured the quantity (to the nearest gram) of all foods (grains, vegetables, meats, etc.) consumed during mealtime. All meat weights were dressed weights after skinning, feathering, and cleaning had taken place. All weigt measurements were carried out using an EatSmart Precision Pro Digital Kitchen Scale, calibrated with a Chrome 100g calibration weight to assure proper scale functioning during the visits. The assistant recorded the individual IDs of all household members, as well as the number, age, and sex of all household guests present during each meal to account for variation in household attendance at meal times. Allocating the weight of household meals to individuals will follow procedures created in past work from this region (22). For food consumption outside of household meals, we collected an individual 24-h recall survey of foods eaten by each household member. For instance, cookies, coffee, honey, alcohol, insects, fruits, etc. that tend to be eaten outside of scheduled meals are accounted for in these six households by surveying each individual once per week throughout the duration of the study.

#### Fisheries Activities

We assessed intra-annual seasonal differences in fisheries catch through weekly shore-side catch surveys, monthly household recall surveys, and the seasonal mapping of fishing ranges. At each of the five sites, we conducted the catch surveys of all fisherman, once per week, following established methodological procedures [see (25)]. During these surveys we recorded, for each fisherman/team of fishermen sighted, the time they spent fishing (time left shore, time returned to shore, and tidal stage), the number of boats in each group, the number of fisherman in each boat, the name of the location where they fished and distance from shore (in minutes), and the method they used for catching seafood (e.g., net, line, spear, trap, hand, or other). Fishermen using nets reported the number and size (mesh size, net length, and net height) of all nets used during that outing. Those using lines, spears, and/or aquatic traps also reported line length, weight, hook size and style, spear style, and/or the number and style of traps used, respectively. A research assistant from the community measured the number of all seafood species (including species of marine invertebrates, fish, reptiles, and mammals; hereafter referred to as “fish”) caught by each fisherman or team of fishermen, the total weight sold, and the total weight intended to be eaten by that fisherman's family. For each individual species of fish caught, we also recorded the total number of that species, their total weight, grams sold, grams eaten by fisherman's family/household, and the cost of one individual of that species of fish on that given day. For five specimens of each species caught by that fisherman on that day, we also measured individual fish length (recorded in centimeters) and weight (grams).

We completed monthly recall surveys of the number of over 75 species of seafood that household members ate during the prior week. We recorded whether each species eaten by that household was caught by a household member, received as a gift, and/or purchased. If caught, households specified both the method used to capture that fish and the distance of capture from the household (in minutes). If purchased, households specified the cost per unit. During these interviews, we recorded the total amount of money earned from the sale of seafood, the percentage of seafood that was sold locally or exported, their total time invested in fishing and indirect fishing-related activities (e.g., net repair), and the total amount spent on all fishing related activities (e.g., materials and/or services) during the prior month.

#### Clinical Visits and Biological Samples

Following procedures from past MAHERY cohorts (18), we conducted the same protocols for two clinical visits (August/September 2016 and November/December 2016), each separated by 2–3 months to account for two distinct fishing seasons (cold/wet and hot/dry, respectively). The cold/wet season corresponds to rough seas and less fish catch and consumption. The hot/dry season corresponds to smoother seas and high fish catch and consumption, often driven by the seasonal flow of pelagic fish into the area. Clinical visits were defined as a consultation with a doctor and the collection of blood and/or other biological samples. The household was notified ~1 week in advance of their scheduled date. Every household visited our health center in their respective community. On the evening prior to the subject's blood draw, each individual scheduled for the following morning came to the health centers etablished for this study to have their height and weight measured and to answer all of the questions in our health survey. We did this to streamline activities the following morning and to remind all individuals that they needed to fast prior to their blood draw. The health survey comprised several questions concerning morbidity recalls, bednet usage, vitamin intake, and medication usage (including deworming medicine). Women of reproductive age were asked about pregnancy and breastfeeding. For anthropometry, we used an ETEKCITY electronic personal digital scale to measure body weight. We measured small children in their mother's arms and then subtracted the mother's independent weight. We measured height using a Seca Road Rod and infant length using a Quick Medical Starters Measure Mat, mid-upper arm circumference (MUAC) of all children 5 years of age and under and the cranial circumferences of all children 2 years of age and under. These anthropometric measurements were collected every 3 months outside of the clinical visits as well.

The following morning, ~24 individuals (typically belonging to 2–6 households) arrived at ~4:15–5:00 a.m., a time chosen to ensure subjects were in a fasting state, as well as to not interfere with the busy daily schedules of the subjects. When subjects arrived, we applied a 5% lidocaine topical anesthetic cream to the area where the needle would be injected to minimize the amount of discomfort from blood collection. We drew blood via venipuncture using 21Gx1.5″ safety needles that was collected into S-Monovette® (Sarstedt, North Carolina) venous blood collection tubes (7.5 ml 15 × 92 mm, Lithium Heparin). Smaller children and infants would have their blood drawn using 23Gx1.5″ safety needles. All blood collection materials were designed for trace metal analysis. Once blood was collected, we inverted tubes three times to properly activate the lithium heparin and attached a Haemo-Diff® with a smear edge so that we could apply a drop of blood onto a slide to create a thin blood smear for microscopy. Another blood drop was applied to a rapid diagnostic test (several brands all provided freely by the Malagasy Ministry of Health) via a capillary tube for immediate malaria diagnosis. A blood drop was then inserted into a HemoCue Hb 201 microcuvette via a capillary tube to test for levels of hemoglobin. Finally, several blood drops were applied on Whatman filter paper FTA cards (two spots per individual) and one drop on OmegaQuant filter paper treated with HUFASave™. Dried blood from the Whatman FTA was used for DNA preservation/extraction and genotyping of Plasmodium infections, among other disease analyses. Dried blood from OmegaQuant filter paper was used to characterize fatty acid profiles for each individual following established protocols (26). Two health care professionals from the local Maroantsetra hospital worked simultaneously on all blood draws. All blood tubes were stored in the dark inside portable refrigerators and kept at 5°C prior to centrifugation. Within 25 min of drawing, all tubes were spun in the centrifuge.

Centrifugation of the lithium heparin tubes permitted the separation of plasma from pelleted red blood cells following centrifugation. We centrifuged tubes at 3,300 RPM for 10 min using the Block Scientific Octafuge Plus Centrifuge (Block Scientific, Inc., Bellport, NY). Following centrifugation, all plasma was pipetted into 1.8 mL cryo-tubes (also trace metal free). These cryo-tubes were then placed in groups of 2–4 inside a section of nylon pantyhose (which is resistant to degradation in liquid nitrogen) and dropped into a liquid nitrogen tank for flash freezing. We obtained plasma samples from 745 individuals over the course of the study. We were unable to obtain venous blood samples from some of the individuals due to unwillingness (*n* = 29) and technical difficulties (*n* = 5), the latter primarily from unsusccessful attempts to find a vein (Figure 1).

All frozen plasma was shipped on dry ice from Madagascar to the Western Human Nutrition Research Center (WHNRC/USDA) via World Courier. At the WHNRC, aliquots of plasma were analyzed for zinc, copper, and iron by inductively coupled plasma-atomic emission spectroscopy (ICP-AES); retinol, β-carotene, α-tocopherol using high performance liquid chromatography with diaode array detection (HPLC-DAD); and vitamin B12, ferritin, transferrin receptors, and inflammation markers (C-reactive protein and α-1-acid glycoprotein) by automated bioanalyzers (Roche e411 and Integra 400).

Fecal samples were provided by each individual enrolled in the study on roughly the same day as clinical visits so that our team could assess the presence and levels of intestinal parasites. Each subject was given a sterile polypropylene screw cap feces collection tube (Sarstedt, Sparks, NV; ref. 80.623). The subject was instructed to defecate on top of the waxy side of a banana leaf and collected three small spoonfuls of feces, which were transferred into the collection tube. Once the samples were returned to our local research team (typically 10 min to 10 h after collection), we added 3 mL of 97% ethanol. All samples were kept at ambient temperature prior to being stored in a −23°C freezer within 14 days of collection, and then shipped on dry ice before being stored at −80°C after shipment to the Harvard T.H. Chan School of Public Health.

At one time point in March 2017, we administered a finger prick to evaluate the prevalence of malaria using a rapid diagnostic test. This permitted analysis of malarial infection across all three seasons in northeastern Madagascar. We also collected fingernail samples at this time. The index finger of the left hand was preferentially used, though sometimes multiple fingers were used to collect an adequate sample. Fingernails of each individual in the study were clipped and weighed on American Weigh Signature Series Digital Pocket Scales (American Weigh Scales, Atlanta, GA) to obtain ~30 mg of fingernails. These fingernails were then placed into a Staples #1 Coin Envelope and folded closed without using the glue seal to avoid contamination. These fingernails will be tested for their carbon and nitrogen isotopic signature as well as for mercury and arsenic content.

## Initial Results

We found a high prevalence of stunting, wasting, and underweight throughout the study population (Table 1). The population is heavily left-skewed with more than 50% of the population being ≤16 years of age, indicating rapid population growth in this region. Households are generally very poor, though far wealthier than non-coastal geographically adjacent populations in the Makira Natural Park (18). Laboratory analyses are still in progress; baseline point-of-care results are shown in Table 1.

**Table 1:** Subject population description of key variables and outcomes.

**Variable**	**Outcome**
Sex (% female)	49.4
Age (median years; min-max)	16.0; (0.1–91.0)
HOUSEHOLD MEDIAN ANNUAL INCOME (CURRENT INTERNATIONAL DOLLARS)^*^	
All communities	$6,840
Community 1	$4,940
Community 2	$6,580
Community 3	$6,590
Community 4	$7,910
Community 5	$4,430
STUNTING AMONG CHILDREN ≤ AGE 5 (% SEVERE; TOTAL)^**^	
Both sexes (*n* = 184)	23.9; 44.2
Females (*n* = 99)	18.2; 37.4
Males (*n* = 85)	28.2; 51.9
UNDERWEIGHT AMONG CHILDREN ≤AGE 5 (% SEVERE; TOTAL)^**^	
Both sexes (*n* = 184)	2.7; 19.6
Females (*n* = 99)	2.0; 14.1
Males (*n* = 85)	3.7; 25.9
WASTING AMONG CHILDREN ≤ AGE 5 (% SEVERE; TOTAL)^**^	
Both sexes (*n* = 184)	2.2; 3.6
Females (*n* = 99)	1.0; 2.0
Males (*n* = 85)	3.7; 6.2
Reproductive aged women (women ages 15–49 as % of all women, *n* = 209)	46.7
Pregnant women (% of women of ages 15–49, *n* = 9)	4.7
Lactating women (% of women of ages 15–49, *n* = 27)	13.4

* *Current international dollars adjusted for purchasing power parity*.

** *Stunting, underweight, and wasting all based on WHO Multicentre Growth Reference Study Group (27)*.

## Discussion

The main strength of this cohort is its detailed longitudinal dietary data that correspond to a suite of nutritional biomarkers and disease targets. The study was purposefully designed to understand the nutritional contribution of seafood to people living in isolated, seafood-dependent regions of Madagascar—a context comparable to many other areas of sub-Saharan Africa and small island developing states of the Pacific (2), particularly in the context of rising metabolic disease and hypertension indicated in Madagascar (28). Another strength of the study is that we sampled a population of both sexes and all ages that was randomized within communities to determine whether impacts were consistent across demographic characteristics, socio-economic status, and fisheries governance regimes (2).

A primary weakness of the study is that the duration (28 months) and observational nature is insufficient to understand the causal relationship between fisheries governance strategies, fish catch, and subjects' nutritional or health status. However, we can create scenarios of the impacts of fisheries governance on fish catch by assuming that the scientific basis for marine conservation is valid. For instance, a global meta-analysis of hundreds of marine protected areas around the world found roughly 1.6 times greater fish biomass in marine protected areas than in unprotected areas (29). We can also use seasonal variation in fish catch to infer the effects of reduced productivity of fisheries in the future.

## Data Availability

All point-of-care health results (anemia and malaria) were provided by our team's health professionals to the study subjects including access to free treatment and referrals to local clinics. All health results from laboratory analyses that were conducted outside of the study area were returned to participants. The health data collected through this study will not be made open access for public use but collaboration is welcomed. Please contact Dr. Christopher Golden (golden@hsph.harvard.edu) for more information.

## Ethics Statement

All households were recruited and enrolled, and each individual consented or assented, following our IRB approved study (Protocol #15-2230, Committee on the Use of Human Subjects, Office of Human Research Administration at the Harvard T.H. Chan School of Public Health). Consent forms were read by literate study members and our team read the form contents to illiterate studymembers. After a discussion of the study materials, consent and permission were attained for all participants. The study was also reviewed and approved by the Malagasy Ministry of Health, and conducted in concert with the regional medical inspector of the Maroantsetra region of Madagascar. Study subjects were not compensated for their participation. However, the population did receive benefits for participating by having access to healthcare professionals working in their community.

## Author Contributions

CG designed the study, led the field research and data analysis, and drafted the manuscript. CB, BR, EA, GB, HRan, and MV co-led the field research, supporting data collection, database synthesis, and analytical support. GB and BV led the database synthesis, and analytical support. BR, DLH, and SV designed and led the malaria research embedded within the cohort. CB, EK, and JG designed a module of the research on environmental shocks and agricultural and health outcomes. LA, DH, and SS-F analyzed blood samples and supported the study design for nutritional biomarkers. SM supported the study design of the cohort and the analysis. DR and HRam supported the study design, field logistics, health system referrals, permitting, and translation of research into policy. All authors contributed to revising and approving the manuscript.

### Conflict of Interest Statement

EK was employed as a graduate student at Cornell University at time of study and writing, and has since moved to Cooper/Smith. This has no impact on the research conducted. The remaining authors declare that the research was conducted in the absence of any commercial or financial relationships that could be construed as a potential conflict of interest. The handling editor declared a shared affiliation, though no other collaboration, with one of the authors CB.
